# Supplementary material for: Spatially resolved random telegraph fluctuations of a single trap at the Si/SiO2 interface
Source: Proc Natl Acad Sci U S A. 2024 Oct 24;121(44):e2404456121. doi: 10.1073/pnas.2404456121 (PMC11536084; doi:10.1073/pnas.2404456121)
Supplement: Supplementary file 1 — Appendix 01 (PDF) [file pnas.2404456121.sapp.pdf]

Supplemental material

for

Spatially resolved random telegraph fluctuations of a single trap at the Si/SiO$_2$ interface

Megan Cowie, Taylor T.Z. Stock, Procopios C. Constantinou, Neil J. Curson, and Peter Grütter

S.1: Distance dependence of donor-like defect noise

The slow $(\mathrm{ms} - \mathrm{s})$ two-state noise measured above donor-like traps cannot be attributed to tunneling between the tip and sample, since noise with similar timescales is measured even when the tip-sample separation is increased (i.e. "tip lifted") by several nanometers, as shown in Figure S1. (Note that the noise amplitude in the Δf channel is very small as compared to the background – i.e. $\sim (1\ \mathrm{Hz})/(500\ \mathrm{Hz}) \approx 0.2\%$, which is why it is indistinguishable in Figure S1a. In the F_d channel (Figure S1b), however, the noise amplitude is comparably large – i.e. $\sim (80\ \mathrm{meV/cycle})/(300\ \mathrm{meV/cycle}) \approx 25\%$.) If the slow noise were due to tunneling between the tip and sample, the noise timescale would be expected to increase significantly as tip lift increased.

In this measurement, this tip acts as a spatially localized gate, meaning that the electric field

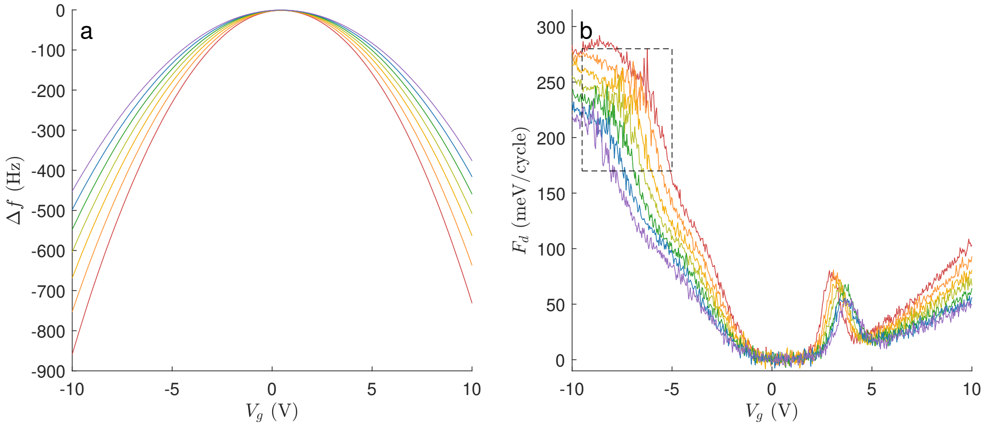

Fig. S1. Distance-dependent bias spectra. Bias spectra near a donor-like trap (seen at negative bias) and an acceptor-like trap (seen at positive bias) at variable tip lift (red $= 0$ nm, purple $= 9$ nm, in steps of 1 nm). The dashed rectangle indicates noise due to the donor-like trap. (The acceptor-like trap does not exhibit additional noise compared to the background.)

at a trap site depends on the spatial separation between the trap and the tip (in x, y, and z). Therefore, as the tip-sample separation increases, we expect the peak noise amplitude to shift to a more extreme bias. Looking at Figure 4 in the main text, increasing the tip-sample separation would effectively shift the upper x-axis (V_g); the lower x-axis ($E_F - E_V$), which is found by applying the MIS capacitor model for a given closest tip-sample separation at variable V_g, would remain unchanged. In this work, the modelled result for the closest tip-sample separation, found by fitting $\Delta f(V_g)$ curves at variable closest tip-sample separation, is $12\ \mathrm{nm}$. While not measured here, the tip-sample separation can also be determined directly by taking a force-distance curve.

The oscillation amplitude is related to the width of the $F_d(V_g)$ peaks above a trap site: As the oscillation amplitude increases, the peak width broadens (see supplementary material for DOI:10.1103/PhysRevLett.132.256202). The bias-dependent two-state noise amplitude measured in this work is expected to exhibit a corresponding broadening, though the noise frequencies are not expected to exhibit a clear amplitude-dependent trend.

S.2: Two-state noise analysis

The methodology to assign states 0 and 1 for an RTS is demonstrated in Figure S2. First, the signal is subtracted from its running mean (with an averaging timescale much longer than the RTS noise timescale). $\Delta(\Delta f)$ and ΔF_d are the difference between Δf or F_d, respectively, and their running means. The subtracted signals are offset so that the 0 state occurs at 0 (Figure S1a,d). Two Gaussian distribution functions are then fitted to histograms of the $\Delta(\Delta f)$ and ΔF_d time traces (Figure S1b,e). The shaded regions in Figure S2a,d show the standard deviation of these Gaussian fits.

States 0 and 1 are assigned by comparing the RTS time traces to the Gaussian fits. First, the first data point is identified as being in the "up" state (0 for Δf and 1 for F_d) or the "down" state. Then, each data point is compared to the previous one. The "up" state flips to "down" if the value is less than the "down" shaded region maximum, and the "down" state flips to "up" if the value is greater than the "up" shaded region minimum. The circled points (Figure S2a,d) show these state flips. The arrows point to mismatches in the Δf and F_d flips; these appear to be state assignment errors, rather than non-correlation between the Δf and F_d RTSs. Specifically, the F_d RTS amplitude is larger than the intrinsic noise (Gaussian peak width), and the flip values are approximately normally distributed in each state (Figure S2f). The Δf RTS amplitude is small compared to the intrinsic noise, and the flip values are not normally distributed (Figure S2c). This indicates an overestimation of Δf flips, and manifests as shorter Δf RTS timescales.

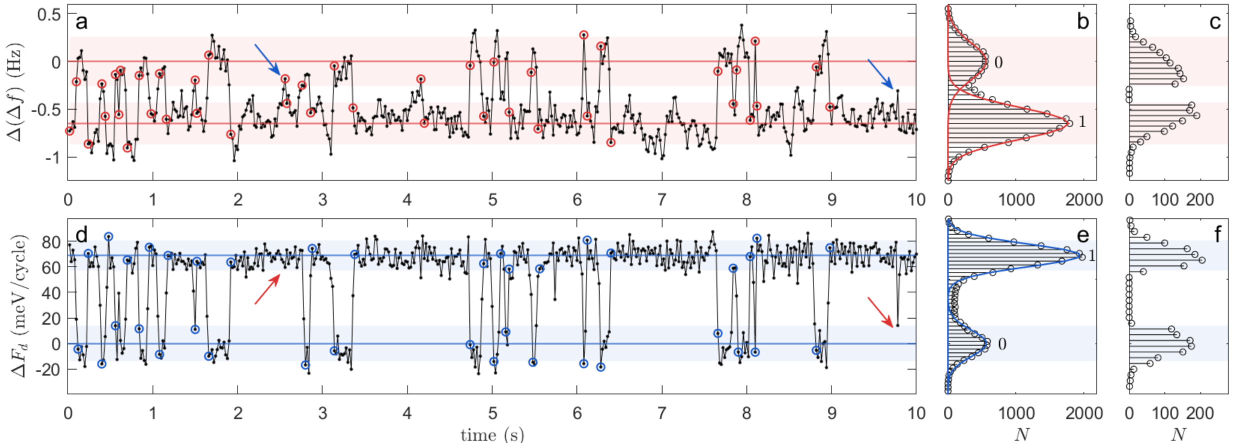

Fig. S1. RTS state assignment methodology. RTS of a donor-like defect at the Si/SiO_2 interface measured at $V_g = -6.5$ V. The timescales found using the methodology described above are: $\tau_0^{\Delta f} = 0.093 \pm 0.007$ s, $\tau_1^{\Delta f} = 0.28 \pm 0.014$ s, $\tau_0^{F_d} = 0.10 \pm 0.01$ s, $\tau_1^{F_d} = 0.33 \pm 0.02$ s.
